# Unveiling intra-population functional variability patterns in a European beech (*Fagus sylvatica* L.) population from the southern range edge: drought resistance, post-drought recovery and phenotypic plasticity

**DOI:** 10.1093/treephys/tpae107

**Published:** 2024-08-20

**Authors:** David Sánchez-Gómez, Ismael Aranda

**Affiliations:** Department of Ecology and Forest Genetics, Instituto de Ciencias Forestales (ICIFOR-INIA), Consejo Superior de Investigaciones Científicas (CSIC), Carretera La Coruña Km 7.5, E-28040 Madrid, Spain; Department of Ecology and Forest Genetics, Instituto de Ciencias Forestales (ICIFOR-INIA), Consejo Superior de Investigaciones Científicas (CSIC), Carretera La Coruña Km 7.5, E-28040 Madrid, Spain

**Keywords:** intra-specific variation, isohydricity, leaf traits, photosynthesis, stomatal conductance, water deficit

## Abstract

Understanding covariation patterns of drought resistance, post-drought recovery and phenotypic plasticity, and their variability at the intra-population level are crucial for predicting forest vulnerability to increasing aridity. This knowledge is particularly urgent at the trailing range edge since, in these areas, tree species are proximal to their ecological niche boundaries. While this proximity increases their susceptibility, these populations are recognized as valuable genetic reservoirs against environmental stressors. The conservation of this genetic variability is critical for the adaptive capacity of the species in the current context of climate change. Here we examined intra-population patterns of stem basal growth, gas exchange and other leaf functional traits in response to an experimental drought in seedlings of 16 open-pollinated families within a marginal population of European beech (*Fagus sylvatica* L.) from its southern range edge. We found a high degree of intra-population variation in leaf functional traits, photosynthetic performance, growth patterns and phenotypic plasticity in response to water availability. Low phenotypic plasticity was associated with higher resistance to drought. Both drought resistance and post-drought recovery of photosynthetic performance varied between maternal lines. However, drought resistance and post-drought recovery exhibited independent variation. We also found intra-population variation in stomatal sensitivity to soil drying, but it was not associated with either drought resistance or post-drought recovery. We conclude that an inverse relationship between phenotypic plasticity and drought resistance is not necessarily a sign of maladaptive plasticity, but rather it may reflect stability of functional performance and hence adaptation to withstand drought. The independent variation found between drought resistance and post-drought recovery should facilitate to some extent microevolution and adaption to increasing aridity. The observed variability in stomatal sensitivity to soil drying was consistent with previous findings at other scales (e.g., inter-specific variation, inter-population variation) that challenge the iso-anisohydric concept as a reliable surrogate of drought tolerance.

## Introduction

Understanding trees’ adaptive potential to environmental stressors is crucial for accurately predicting the impacts of climate change on forest vulnerability, resilience and ecosystem services (Bussotti and Pollastrini 2021). Standing genetic variation is the main source for rapid adaptation to novel environments (Barrett and Schluter 2008). Complementarily, phenotypic plasticity, the ability of a genotype to express different phenotypes in response to environmental conditions, can broaden phenotypic variability beyond genetic boundaries, leading to rapid phenotypic shifts in few generations (West-Eberhard 1989; Matesanz and Ramírez-Valiente 2019). However, phenotypic plasticity is not necessarily adaptive and empirical evidence of adaptive plasticity is often the exception rather than the rule (Van Kleunen and Fischer 2005; Ghalambor et al. 2007).

Intra-specific variation, which includes both genetically based variability and phenotypic plasticity within a species, plays a significant role in enhancing the adaptive potential and resilience of populations, as well as in shaping the structure and dynamics of ecosystem (Benito Garzón et al. 2011; Des Roches et al. 2018). Marginal populations hold particular significance in this context. Marginal populations are highly vulnerable to environmental unpredictability due to their small size, isolation and positioning at the species’ niche limits (Hampe and Petit 2005; Hampe and Jump 2011; Angert et al. 2020). Yet, these populations are also recognized as reservoirs of valuable genetic resources for stress tolerance (Hardie and Hutchings 2010).

Water deficit is an increasingly significant stress factor in the current climate change scenario, with droughts and rising temperatures already impacting tree growth, survival and recruitment (Allen et al. 2010; Hu et al. 2017; Kueppers et al. 2017; Dannenberg et al. 2019; DeSoto et al. 2020).

Down-regulation of stomatal conductance is a primary mechanism limiting photosynthesis and growth during water deficits. The sensitivity of this process to dehydration can determine different functional strategies with implications for drought tolerance within the iso-anisohydric continuum (Tardieu and Davies 1993; Jones 1998; Tardieu and Simonneau 1998). While interspecific variation along the iso-anisohydric continuum is well documented in trees species (Klein 2014; Meinzer et al. 2016; Salvi et al. 2022), intra-specific variation remains largely unexplored (but see Leuschner et al. 2022).

Trees exhibit drought tolerance in various ways. They can maintain functional performance during water deficit (drought resistance), they can recover functional performance after drought (post-drought recovery) or they can do both to varying degrees. Immediate tree physiological responses involved in drought resistance have been widely studied. In parallel, information on delayed physiological responses (such as post-drought recovery) is also accumulating (e.g. Kirschbaum 1988; Marron et al. 2003; Gallé et al. 2007; Cano et al. 2014; Perez-Martin et al. 2014; Pšidová et al. 2015; Arend et al. 2016; Hájíčková et al. 2017; Hesse et al. 2023). Yet, a complete understanding of the association between drought resistance and post-drought recovery remains elusive. For example, a direct association between drought resistance and post-drought recovery in cool-season grasses (Taleb et al. 2023) suggests that it is possible for specific plant groups to evolve maximizing both drought resistance and post-drought recovery mechanisms. However, in trees, an inverse relationship between drought resistance and post-drought recovery has been found, which could be attributed to differences in the xylem anatomy of the studied species (Vanhellemont et al. 2019). Similarly, an inverse relationship between growth resistance to drought and recovery has been observed at large regional scales that include a wide range of forest species (Gazol et al. 2017) suggesting the potential existence of a functional trade-off.

European beech (*Faguls sylvatica* L.), hereafter beech, is a drought-sensitive forest tree species (Ellenberg 1992; Aranda et al. 2005; Gallé and Feller 2007; Cavin et al. 2013; Leuschner 2020) with high ecological and socio-economic importance in Europe (Martinez del Castillo et al. 2022). This species has endured growth declines and mortality events at local and regional scales attributed to heat waves and drought (Knutzen et al. 2017; Leuschner 2020). According to climate change projections, these impacts may escalate in extent and severity, which could lead to further declines in growth rates and result in strong range contractions (Saltré et al. 2015). These impacts may be particularly pronounced at its southern range edge (Martinez del Castillo et al. 2022) where drought is a primary limitation (Gutiérrez 1988; Dittmar et al. 2003).

Droughts can have severe impacts at early ontogenetic stages when trees are most vulnerable (Harper 1977), affecting regeneration and tree recruitment (Muffler et al. 2021), critical for beech persistence (Silva et al. 2012; Pozner et al. 2022). Despite long-standing information on inter-population variability in the functional response of beech to water limitation (Tognetti et al. 1995; Rose et al. 2009; Sánchez-Gómez et al. 2013; Knutzen et al. 2015; Stojnić et al. 2018), data on intra-population variation in response to water limitation are scarcer (but see Aranda et al. 2017, 2018). Furthermore, the potential of phenotypic plasticity to increase drought tolerance, particularly at the intra-population level, remains poorly understood. Understanding intra-population functional variation in response to water stress of beech seedlings at its southernmost range edge is crucial for anticipating the vulnerability of these populations to increased droughts and more generally, for assessing the species’ adaptive potential in a drier Europe.

This study investigates the functional response of beech seedlings to experimental water deficit across 16 open-pollinated families from a marginal population in south-western Europe. We analyse intra-population variability of phenotypic plasticity to water availability, post-drought recovery of photosynthetic performance, and their associations with stem growth and other leaf functional traits. Our experimental hypotheses were: (i) phenotypic plasticity in response to water availability differs among beech families and is linked to improved physiological performance under water stress, (ii) post-drought recovery varies among families, with recovery associated with plasticity and enhanced physiological performance under water stress, and (iii) stomatal sensitivity to soil drying varies at the intra-population level and is associated with increased photosynthetic performance under drought. To test these hypotheses, we performed new analyses on data from a previous study (Aranda et al. 2017). We also include new data on post-drought recovery of photosynthetic performance. More precisely, as a novelty with respect to the previous work, we calculated indices of phenotypic plasticity and post-drought recovery, analysed their variability, and examined their covariation with other functional traits. Additionally, we provide new analyses on stomatal conductance sensitivity to soil drying and its variability among beech families.

## Materials and methods

### Experimental setting and functional traits analysed

We used beech seedlings of 16 strictly open-pollinated families from ‘Montejo de la Sierra’ beech-forest (41°7′N, 3°30′W, Madrid, central Spain). Beechnuts were collected from 24 families; however, only 16 families produced a sufficient number of seedlings for the experiment. A total of 320 1-year-old seedlings (5 replicates × 16 families × 2 watering regimes × 2 blocks) were grown and arranged in a split-plot design within a greenhouse, with ‘Family’ and ‘Watering Treatment’ as the main experimental factors (Fig. S1 available as Supplementary data at *Tree Physiology* Online). Well-watered (WW) seedlings were watered three times a week. This protocol maintained soil volumetric water content of WW-seedlings above 20% throughout the experiment (Fig. S2 available as Supplementary data at *Tree Physiology* Online). Water-stressed (WS) seedlings were submitted to 11 consecutive weekly cycles of water stress. Each cycle accounted for 6 days of water deprivation followed by pot soil rehydration to the soil water holding capacity on the seventh day (Fig. S1 available as Supplementary data at *Tree Physiology* Online). The imposed water stress did not result in any mortality events. Gas exchange along with soil volumetric water content, and predawn water potential measurements, were taken at the end of the water deficit period (right after the 11th cycle of water stress). Gas exchange and chlorophyll fluorescence were measured with a portable photosynthesis system (LiCor 6400 XP, Li-COR Inc., Lincoln, NE, USA) coupled to an integrated fluorescence chamber (chamber Li-6400-40, Li-COR Inc.). We also measured relative growth rate (RGR) of stem basal area at different time intervals throughout the experiment measuring the collar diameter (±0.01 mm) with a digital caliper. The measurement point was marked with a permanent marker so that subsequent measurements could be made at the same point. We considered three time intervals *T1* (from 1st to 5th cycle of water stress), *T2* (from 5th to 11th cycles of water stress) and *Tot* (from 1st to 11th cycle of water stress) for this purpose. Leaf samples for elemental nitrogen and carbon, stable isotopic fractioning for carbon and nitrogen and specific leaf area determination were also sampled right after the 11th cycle of water stress. The functional traits analysed are described in Table 1. See also Aranda et al. (2017) for additional details.

**Table 1:** Description of the studied functional traits.

**Trait**	**Description**
A_area_ (μmol m^−2^ s^−1^)	Net photosynthesis on an area basis (gas exchange trait)
A_mass_ (nmol g^−1^ s^−1^)	Net photosynthesis on an mass basis (gas exchange trait)
g_wv_ (mol m^−2^ s^−1^)	Stomatal conductance to water vapour (gas exchange trait)
iWUE (μmol mol^−1^)	Intrinsic water-use efficiency defined as the ratio of A_area_ to g_wv_ (gas exchange trait)
PNUE (μmol g^−1^ s^−1^)	Leaf photosynthetic nitrogen-use efficiency
Φ_PSII_	Actual photochemical efficiency of photosystem II (determined by chlorophyll fluorescence)
SLA (m^2^ kg^−1^)	Specific leaf area (leaf area per unit of leaf biomass)
N_m_ (%)	Elemental leaf nitrogen content
C_m_ (%)	Elemental leaf carbon content
δ^13^C (‰)	Stable leaf carbon isotope ratio
δ^15^N (‰)	Stable leaf nitrogen isotope ratio
Ψ_pd_ (MPa)	Predawn water potential (assessment of plant water status)
RGR	Basal area relative growth rate; this was determined at three time intervals throughout the experiment (*T1*, *T2* and Tot, see the main text)

After the 11th cycle of water deficit WS-seedlings were allowed to recover from water stress (Fig. S1 available as Supplementary data at *Tree Physiology* Online). The same watering protocol in WS-seedlings as in WW-seedlings was applied for two additional weeks during post-drought recovery. Finally, gas exchange measurements were repeated after this recovery period.

### Statistical analyses

We used analysis of variance (ANOVA) to test for the significant effect of the main factors (“Family’ and ‘Watering Treatment’) and their interaction on gas exchange traits after recovery from water stress. Shapiro–Wilk and Levene’s tests were used to check for normality and homoscedasticity, respectively.

A log-linear model describing the response of g_wv_ as a function of Ψ_pd_ and the factor ‘family’ was also analysed for measurements at the end of the water stress period. The model can be described as:


(1) \begin{equation*} {-} Ln\ \left({y}_{ij}\right)={\alpha}_0+{\alpha}_i+\left({\beta}_0+{\beta}_i\right)\ {x}_{ij}+{\varepsilon}_{ij}, \end{equation*}

where ${y}_{ij}$ is the *g_wv_* value of the *j*th observation of the *i*th family, ${\alpha}_0$is the common intercept, ${\alpha}_i$ is the increment of the *i*th family to the common intercept, ${\beta}_0$ is the common slope, ${\beta}_i$ is the increment of the *i*th family to the common slope, ${x}_{ij}$ is the Ψ_pd_ value of the *j*th observation of the *i*th family and ${\varepsilon}_{ij}$ is the error term.

To analyse inter-familiar variation in phenotypic plasticity to water availability and post-drought recovery of gas exchange traits, we estimated an index of phenotypic plasticity to water availability (PP) and an index of post-drought recovery (RE). We applied the relative distance plasticity index (RDPI) of Valladares et al. (2006) for both PP and RE. PP and RE were calculated for each functional trait and family as:


(2) \begin{equation*} \mathrm{Index}\ \left( PP\ \right|\ RE)=\sum \left({d}_{ij}\to{i}^{\prime }{j}^{\prime }/\left({x}_{i^{\prime }{j}^{\prime }}+{x}_{ij}\right)\right)/n,i\ne{i}^{\prime } \end{equation*}

where ${d}_{ij}\to{i}^{\prime }{j}^{\prime }$is pair-wise distance or difference between the observations ${x}_{i^{\prime }{j}^{\prime }}$ and ${x}_{ij}$, *i* identifies the treatment level and *j* identifies the observation number within the treatment. The difference between these indices is that PP was calculated for traits measured at the end of the water stress period while RE was calculated for traits measured at the end of the recovery period. These indices range from 0 (absence of plasticity for PP and complete recovery for RE) to 1 (maximum plasticity for PP and absence of recovery for RE). Differences among families in PP and RE indices were tested following (Valladares et al. 2006). PP and RE indices estimated for each functional trait were subjected to hierarchical clustering to arrange the observed variability according to similarity of the estimated indices across beech families. Data normalization was not required because PP and RE indices were already scaled, ranging from 0 to 1.

We also performed a principal components analysis (PCA) including PP and RE indices and the values of the functional traits split into well-watered and water-stressed conditions.

Finally, bivariate correlations between all the variables included in PCA were also analysed. Significance level of the correlation matrix was FDR-corrected.

All the statistical analyses were done with R (R Core Team 2023). Analyses of linear models and Shapiro–Wilk’s test were analysed with functions *lm*, *aov* and *shapiro.test* in the ‘stats’ package (R Core Team 2023). Levene’s test was done with *levene.test* function in ‘car’ package (Fox and Weisberg 2019). Heatmap plotting and hierarchical clustering were performed with functions *heatmap.2*, *hclust* and *pvclust* in ‘gplots’ (Warnes et al. 2022), ‘stats’ and ‘pvclust’ (Suzuki et al. 2019) packages, respectively. PCA analysis was performed with functions *prcomp* and *autoplot* in ‘stats’ and ‘ggfortify’ (Horikoshi and Tang 2016; Tang et al. 2016) packages, respectively. Bivariate correlations were analysed and plotted with function *corrplot* in ‘corrplot’ package (Wei and Simko 2021).

## Results

### Recovery from water stress and phenotypic plasticity to water availability

**Figure 1:**
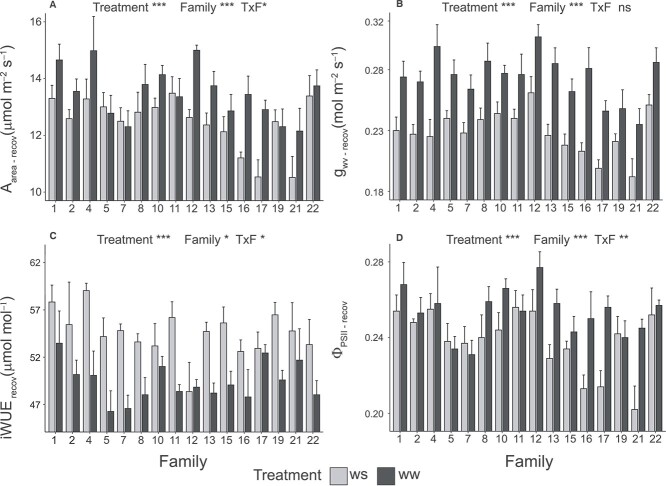
Gas exchange traits for each beech family and watering treatment right after the recovery period. Significance of the effect of the watering treatment, family and the interaction between both—T×F—are shown for each trait. ^*^P < 0.05, ^*^^*^P < 0.01, ^*^^*^^*^P < 0.001, ns *P* > 0.05.

Post-drought recovery of gas exchange traits differed among beech families. The effect of treatment (*T*) and family (*F*) was significant for all the studied gas exchange traits while the interaction term *T×F* was significant for all traits but g_wv_ (Fig. 1). While some families had a good or complete recovery of A_area_ and Φ_PSII_, poor recovery was found in general for g_wv_ (Fig. 1, Table S1 available as Supplementary data at *Tree Physiology* Online). Accordingly, iWUE remained higher (Fig. 1) in recovered seedlings from water stress (WS) than in the control group (WW).

**Figure 2:**
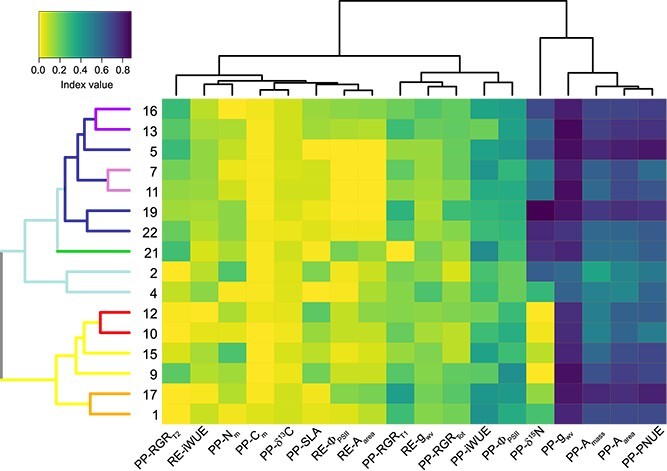
Heat map for phenotypic plasticity and recovery indices. Includes cluster dendrogram for the factor ‘family’ and estimated indices. Branches with different colours highlight familial clusters with high support *P* < 0.05 after 1000 boostraping computations. PP or RE before the name of the trait denotes either phenotypic plasticity, or post-drought recovery indices for the corresponding trait.

Beech displayed different degrees of phenotypic plasticity (PP index) to water availability depending on the functional trait considered. For example, g_wv_ and other closely related photosynthetic traits such as A_area_, A_mass_ and PNUE had high plasticity values (Fig. 2, Table S1 available as Supplementary data at *Tree Physiology* Online). In contrast, leaf carbon and nitrogen content, δ^13^C and SLA had low plasticity values (Fig. 2, Table S1 available as Supplementary data at *Tree Physiology* Online). Phenotypic plasticity differed also among families with significant inter-familial variation found for all the studied traits (Table S1 available as Supplementary data at *Tree Physiology* Online). Beech families were grouped according to similarity in plasticity and recovery patterns. Two large distinct clusters were found. The first one included the families 1, 17, 9, 15, 10 and 12, while the second one included the rest of families (Fig. 2). Lower distance clusters with high support within these large clusters were also identified. For example, pairs of families with very similar plasticity and recovery patterns were 16–13, 7–11, 12–10 and 17–1 (Fig. 2).

### Stomatal response to soil drying

We found that g_wv_ increased with Ψ_pd_ following an exponential relationship. Maximum values of g_wv_ were slightly higher than 0.30 mol m^−2^ s^−1^ with high variability found at Ψ_pd_ above −1.2 MPa (Fig. S3 available as Supplementary data at *Tree Physiology* Online). Very low but still measurable g_wv_ was recorded at −3.3 MPa (minimum Ψ_pd_). A significant interaction was found between Ψ_pd_ and the factor family on their effect on g_wv_ denoting that the relationship between these two traits is family dependent (Table 2). In particular, the slope of the log-linear relationship between g_wv_ and Ψ_pd_$\left(\beta \right)$ significantly differed among families. For instance, family 17 displayed the lowest slope while families 7, 10 and 11 had the highest slopes (Table 2).

**Table 2:** Analysis of variance of a log-linear model describing g_wv_ as a function of predawn water potential (Ψ_pd_) and the factor ‘family’. Estimates and confidence intervals of regression coefficients are provided for each beech family. See description of the Model in materials and methods. The parameter $\alpha$ is the intercept of the model for each family (${\alpha}_0+{\alpha}_i$) and $\beta$ is the slope (${\beta}_0+{\beta}_i$) as described in the main text. Letter codes denote homogeneous groups (families with overlapping confidence intervals share the same letter).

Effect	Df	Sum square	Mean square	*F*-value	*P*-value	Family	$\alpha$ ± 95% CI	$\beta$ ± 95% CI
Ψ_pd_	1	265.12	265.12	946.34	**<0.001**	1	0.97 ± 0.57 a	−1.24 ± 0.46 ab
Family	15	3.55	0.24	0.84	0.63	2	0.91 ± 0.40 a	−1.29 ± 0.38 ab
Ψ_pd_ × Family	15	7.45	0.50	1.77	**0.04**	4	0.73 ± 0.48 a	−1.43 ± 0.44 ab
Residuals	275	77.04	0.28			5	1.10 ± 0.42 a	−1.27 ± 0.29 ab
						7	0.74 ± 0.47 a	**−1.46 ± 0.39 a**
						9	0.79 ± 0.39 a	−1.19 ± 0.26 ab
						10	0.80 ± 0.32 a	**−1.44 ± 0.30 a**
						11	0.73 ± 0.42 a	**−1.50 ± 0.34 a**
						12	0.63 ± 0.54 a	−1.27 ± 0.40 ab
						13	0.89 ± 0.39 a	−1.22 ± 0.27 ab
						15	1.11 ± 0.43 a	−1.17 ± 0.33 ab
						16	1.01 ± 0.31 a	−1.19 ± 0.20 ab
						17	1.41 ± 0.49 a	**−0.77 ± 0.29 b**
						19	1.12 ± 0.31 a	−1.16 ± 0.22 ab
						21	1.13 ± 0.56 a	−1.02 ± 0.44 ab
						22	1.06 ± 0.50 a	−1.13 ± 0.44 ab

Significant *p*-values and families with significantly different regression coefficients are highlighted in bold.

### PCA analysis and bivariate correlations

**Figure 3:**
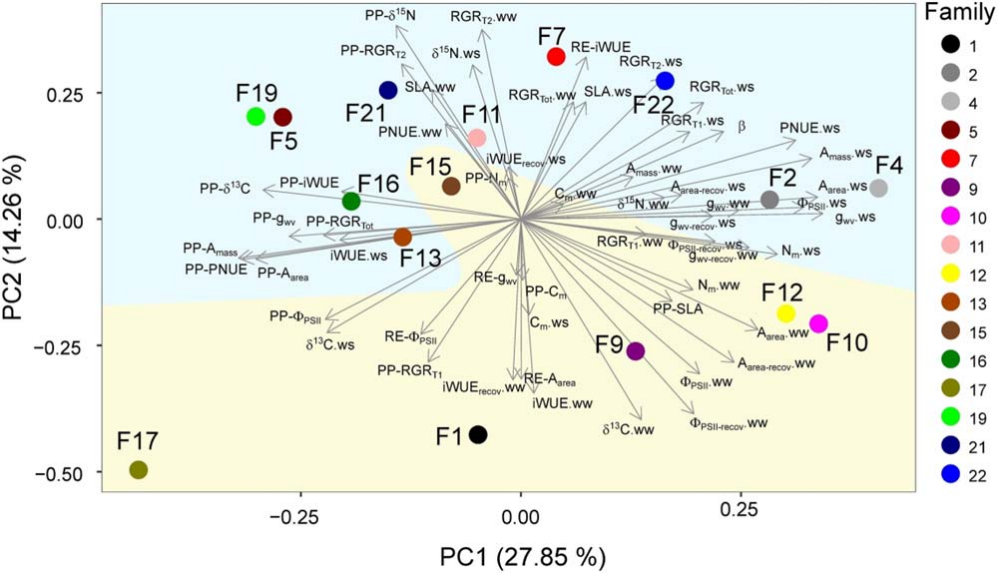
Principal component 2-D biplot displaying factor coordinates of the studied traits and PP- and RE-indices for each beech family. The variance explained by the principal components (PC) is indicated on each axis. β stands for the slope of the log-linear model describing g_wv_ as a function of predawn water potential (Ψ_pd_). The acronyms for the rest of the traits are defined in Table 1. The values of traits are split into watering treatments—well-watered ‘ww’ or water-stressed ‘ws’. The two main family clusters identified in Fig. 2 are highlighted by the two large areas in different colours.

Two principal components (PC) were extracted (eigenvalue > 1), which accounted for 42.11% of the total variance. The traits with the highest positive loadings on PC1 (27.85% of the total variance) were g_wv_, A_area_, A_mass_, Φ_PSII_, PNUE and N_m_ under water-stressed conditions; in contrast, PP-A_mass_, PP-PNUE, PP-A_area_ and PP-δ^13^C were the traits with the highest negative loadings on PC1 (Fig. 3). The traits with the highest positive loadings on PC2 (14.26% of the total variance) were PP-δ^15^N, RE-iWUE and RGR_T2_ under well-watered conditions while RE-A_area_ and δ^13^C, Φ_PSII-recov_, iWUE and iWUE_recov_ under well-watered conditions were the traits with the highest negative loadings on PC2 (Fig. 3). In general, the plasticity of gas exchange traits and their values under water stress occupied opposite extremes along the PC1 axis, indicating an inverse relationship between these variables. In contrast, the loadings of the recovery indices were very low on PC1 but much higher on PC2 (Fig. 3). This suggests an independence between post-drought recovery and both photosynthetic performance under stress and plasticity. Beech families were evenly distributed across the space defined by the two principal component axes. Nevertheless, the two major clusters identified in the analysis of plasticity and recovery indices (Fig. 2) remained largely consistent upon including the rest of variables (Fig. 3). Similarly, pairs of families that defined previously identified lower distance clusters maintained relatively close coordinates in the PCA analysis (e.g., 2–4, 1–17, 7–11 and 10–12).

**Figure 4:**
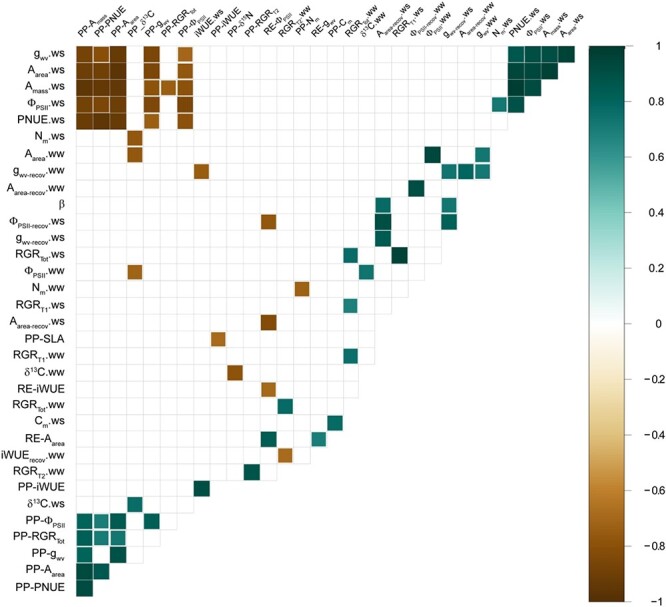
Correlation plot for the studied traits and indices. Non-significant (*P* > 0.05) Pearson’s correlations coefficients ‘r’ are not shown. Rows and columns without any significant correlation were removed. The colour scale represents ‘r’ values ranging from −1 to 1.

**Figure 5:**
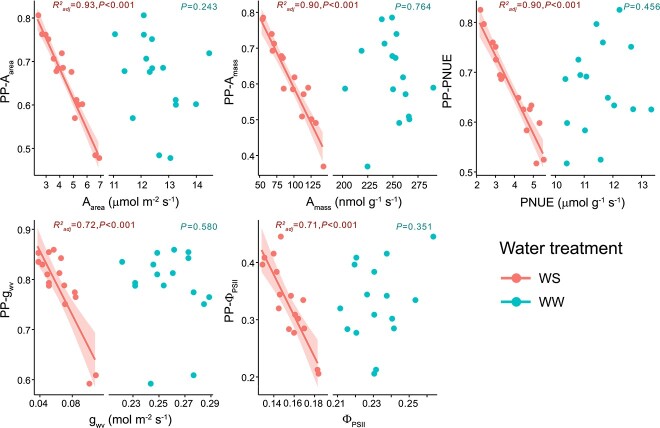
Relationship between plasticity in leaf photosynthetic traits and the corresponding trait values in both water-stressed and well-watered plants**.** Regression lines and confidence bands are shown for significant correlations.

Significant positive bivariate correlations were found for almost all pairs of phenotypic plasticity indices of gas exchange traits. PP-A_mass_ (phenotypic plasticity index for A_mass_) was positively correlated to PP-A_area_, PP-g_wv_, PP-Φ_PSII_ and PP-PNUE (Fig. 4). Similarly, plasticity of RGR_Tot_ (PP-RGR_Tot_) was positively correlated to PP-A_mass_, PP-A_area_ and PP-PNUE (Fig. 4). Analogous correlations with RGR_T1_ or RGR_T2_ followed a similar trend but were not significant (Fig. S4 available as Supplementary data at *Tree Physiology* Online). In contrast, phenotypic plasticity of most traits was negatively correlated to the values of the corresponding trait under water stress (i.e., PP-A_mass_ vs A_mass_-ws, PP-g_wv_ vs g_svw_-ws, Fig. 5), in agreement with their opposite loadings on PC1 (Fig. 3), except for iWUE, δ^13^C and C_m_ (Fig. 4).

Post-drought recovery of gas exchange traits did not correlate with either the phenotypic plasticity of those traits or the corresponding values of the same traits under water stress (Fig. 4, Fig. S5 available as Supplementary data at *Tree Physiology* Online). Finally, no association was found between the slope of the relationship between g_wv_ and Ψ_pd_$\left(\beta \right)$ and photosynthetic performance under water stress (i.e., A_mass-ws_ or A_area-ws_, Fig. 4).

## Discussion

### Phenotypic plasticity

We found intra-population variation in phenotypic plasticity in response to water availability for stem growth and several leaf-level functional traits. Likewise, inter-population variation in phenotypic plasticity in response to water availability or other environmental factors has previously been found in this (Meier and Leuschner 2008; Wortemann et al. 2011; Sánchez-Gómez et al. 2013; Stojnić et al. 2015; Frank et al. 2017; Čortan et al. 2019; Petrík et al. 2020) and other forest tree species (Gratani et al. 2003; Lauteri et al. 2004; Baquedano et al. 2008; Gimeno et al. 2009; Ramírez-Valiente et al. 2010; Corcuera et al. 2011). The expression of phenotypic plasticity is often interpreted in terms of the adaptive potential of the species (Schlichting 1986) but an explicit analysis of the adaptive value of this plasticity or its association with functional performance under different environments is generally lacking, in particular at the intra-specific level (but see Cooper et al. 2019; Petrík et al. 2020; de la Mata et al. 2022). Here we addressed this analysis and found that drought resistance (defined as comparatively high level of photosynthetic performance and stem growth under water stress) was associated with low phenotypic plasticity across families. Similarly, low plasticity has been associated with reduced drought-induced mortality in beech (Petrík et al. 2020). In fact, an increasing number of studies reports low plasticity in association with enhanced plant performance in stressful conditions (Valladares et al. 2000; Sánchez-Gómez et al. 2006; Kreyling et al. 2019; Power et al. 2019; Stotz et al. 2021; Solé-Medina et al. 2022).

These findings have been discussed arguing that a lack of plasticity (canalization of the phenotype) might be the best strategy under resource limitation because in that situation the optimal phenotype is unlikely to be produced by plasticity (Ghalambor et al. 2007) and the cost of plasticity might outweigh its benefit (Pigliucci 2001; Valladares et al. 2007). However, we believe that the most likely explanation for our results is related to the nature of the studied traits. Most of the traits analysed in this study have a strong fitness component with a functional implication in carbon assimilation and growth. In fact, the positive correlation found between plasticity of carbon uptake capacity (PP-A_mass_ and PP-A_area_) and plasticity of growth (PP-RGR_Tot_) indicates that the impact of water stress on carbon uptake is subsequently reflected in plant growth. We argue that traits that can be used as surrogates for fitness, such as most performance traits, should not be plastic when fitness is maintained invariant across environments. In Bonser’s words, ‘plasticity in a given trait can be adaptive if the capacity to express a range of phenotypes in different environments allows plants to maintain fitness across environments’ (Bonser 2021). Therefore, depending on the traits considered both high and low plasticity could reflect adaptation. Following this reasoning, we wonder whether the paucity of evidence on adaptive plasticity (Van Kleunen and Fischer 2005; Ghalambor et al. 2007) might be largely the result of the high percentage of studies that misinterpret the adaptive value of plasticity according to the nature of the traits for which plasticity is assessed (Bonser 2021).

### Stomatal stringency and intra-specific functional diversity

The degree of stomatal stringency in response to dehydration has been associated with the stability of the leaf water status (Tardieu and Simonneau 1998; Klein 2014), distinguishing between isohydric behaviour (i.e., sensitive stomata to dehydration leading to fairly stable leaf water potentials) and anisohydric behaviour (i.e., low sensitive stomata to dehydration leading to pronounced drops of the leaf water potential). Here we found that the sensitivity of stomatal conductance to soil drying differed across maternal lines. Certain families had a more stringent control of water losses than others did, suggesting the degree of isohydricity in beech can vary at the intra-specific level. Earlier reports on the stringency of beech stomatal regulation show significant variability across studies (Oren et al. 1999; Bréda et al. 2006; Köcher et al. 2009; Jonard et al. 2011). This has been mainly attributed to phenotypic variation induced by heterogeneity in soil water availability and air humidity across studies or populations evaluated (Leuschner et al. 2022). In the present study, the environmental factors were controlled and uniform across the studied beech families, so they should not have had a confounding effect on the observed response patterns. According to the results of this study, we argue that intra-population genetic diversity controlling the stomatal sensitivity to soil drying can also contribute to explain the variability of beech’s stomatal behaviour. However, this variability did not apparently have a major influence on leaf level drought resistance and post-drought recovery of the studied beech families. This is consistent with recent evidence on inter-population (Leuschner et al. 2022) and inter-specific (Martínez-Vilalta and Garcia-Forner 2017) variability which shows that a stringent stomatal control is not necessarily associated with narrower ranges of leaf water potentials as previously thought. Therefore, caution should be taken when interpreting iso/anisohydricity or one of its components such as the stringency of stomatal regulation in terms of drought tolerance or drought resistance.

### Post-drought recovery

In general, photosynthetic rates and photochemical function recovered better than stomatal conductance after drought release. Yet we found intra-population variability in the recovery response patterns. Several families fully recovered photosynthetic rates and photochemical performance after 2 weeks of drought release but none of the families recovered stomatal conductance to control values, resulting in iWUE remaining high across families after stress alleviation. Despite the inter-study variability of the time to reach full post-drought recovery of photosynthetic performance (Pflug et al. 2018), a common pattern emerges in beech, pointing to photosynthetic rates recovering faster than stomatal conductance (Tognetti et al. 1995; Gallé and Feller 2007; Sánchez-Gómez et al. 2013; Blessing et al. 2016; Pflug et al. 2018). Likewise, this recovery response pattern has been reported in other woody species (Kirschbaum 1988; Gallé et al. 2007; Liu et al. 2010; Li et al. 2021) but not always, (Cai et al. 2005) suggesting that although it seems to be a widespread response among tree species, it may not be completely generalizable.

The initial increase in iWUE after drought release is probably an ABA-mediated effect on stomatal regulation in response to previous water stress (Kirschbaum 1988). Nevertheless, this effect could be transient until maximum stomatal conductance is fully recovered. Interestingly, the finding of post-drought stimulation of photosynthetic rates but unaltered stomatal conductance after complete recovery (Pflug et al. 2018) invite speculation that, in certain woody species such as beech, increased iWUE can be maintained for long after drought release, even though maximum stomatal conductance is fully recovered. The potential adaptive value and generality of this post-drought stimulation of photosynthesis and sustained increase of iWUE deserves further investigation.

We analysed as well the relationship between drought resistance and post-drought recovery. While it was possible to simultaneously find families with high drought resistance and high post-drought recovery (e.g. family 17), such direct association did not hold across families (i.e. drought resistance and post-drought recovery varied independently across families). This finding suggests that beech’s functional response to water deficit is unlikely to be constrained by a genetic or functional dependence between traits involved in drought resistance and post-drought recovery and contrasts with previous studies supporting a trade-off (Gazol et al. 2017; Li et al. 2020). Yet those studies addressed covariation patterns of drought resistance and post-drought recovery at the inter-specific level.

Although comparative functional studies reveal that beech is in general more vulnerable to droughts than co-occurring tree species (Aranda et al. 1996, 2005; Leuzinger et al. 2005; Cano et al. 2013; Kasper et al. 2022), its high post-drought recovery potential could compensate for its comparatively lower drought resistance (Vanhellemont et al. 2019). Hence, post-drought recovery could be particularly relevant for beech’s resilience and for maintaining tree species coexistence in mixed beech forests under more frequent and severe droughts.

Our findings align with previous research indicating significant intra-population variability in the functional traits (Aranda et al. 2017; Schmeddes et al. 2024) and metabolic patterns (Aranda et al. 2018) of beech. This functional diversity coupled with that observed at the inter-population level (e.g. Meier and Leuschner 2008; Rose et al. 2009; Bresson et al. 2011; Wortemann et al. 2011; Dounavi et al. 2016; Schuldt et al. 2016) suggests that beech possesses substantial local and regional adaptive potential under increasingly arid conditions. Our findings further support the hypothesis that both genetic and epigenetic variability may underlie this adaptive potential as has been proposed for other species (Herrera and Bazaga 2010; Lele et al. 2018). Contrary to the prediction of limited genetic variation in marginal populations (e.g. Hardie and Hutchings 2010), our results indicate that beech populations at the trailing edge exhibit traits indicative of enhanced drought tolerance compared with other populations (Rose et al. 2009; Sánchez-Gómez et al. 2013; Kreyling et al. 2014; Thiel et al. 2014). Moreover, this study supports that this populations can maintain high degrees of intra-population functional variability (Aranda et al. 2017, 2018) contributing significantly to the overall phenotypic variability of the species (Schmeddes et al. 2024).

While our findings are consistent with previous studies on this topic, further research is necessary to determine whether the intra-population functional variation observed in this study is consistent across other populations of the species and to assess the generalizability of these findings.

## Conclusions

In this study, we present evidence of significant intra-population variability in a beech population from the trailing edge in terms of key leaf functional traits and growth patterns in response to water availability. Both genotype and phenotypic plasticity contributed significantly to the observed phenotypic variation. Differences in plasticity among families were associated with differences in functional performance under drought. More precisely, low phenotypic plasticity was associated with improved functional performance under drought, reflecting more stable functional performance and less drought-induced damage. Our research revealed not only in intra-population variability in drought resistance but also in post-drought recovery, with these two functional abilities exhibiting independent variation. Therefore, drought resistance and post-drought recovery may evolve in response to aridity more rapidly than if they were mutually constrained (e.g., in the case of a genetic or functional trade-off), facilitating local adaptation. Stomatal sensitivity to soil drying differed between maternal lines but this was not significantly associated to drought resistance or post-drought recovery. This is consistent with previous findings at other scales (e.g. inter-specific variation, inter-population variation) that challenge the iso-anisohydric concept as a reliable surrogate of drought tolerance. The observed variability in beech’s post-drought recovery may be of particular importance. The drought resistance of beech is generally lower than that of co-occurring species. Therefore, its recovery potential, rather than its drought resistance, may be the main determinant of its competitive ability in future scenarios of increased aridity.

## Supplementary Material

supplementary-dsg-ia-revision_tpae107

## Data Availability

Data will be available upon request to the corresponding authors.
