# Supplementary material for: Unveiling intra-population functional variability patterns in a European beech (Fagus sylvatica L.) population from the southern range edge: drought resistance, post-drought recovery and phenotypic plasticity
Source: Tree Physiol. 2024 Aug 20;44(9):tpae107. doi: 10.1093/treephys/tpae107 (PMC11412075; doi:10.1093/treephys/tpae107)

Supporting Information

Article title: UNVEILING INTRA-POPULATION FUNCTIONAL VARIABILITY PATTERNS IN A EUROPEAN BEECH (*FAGUS SYLVATICA* L.) POPULATION FROM THE SOUTHERN RANGE EDGE: DROUGHT RESISTANCE, POST-DROUGHT RECOVERY, AND PHENOTYPIC PLASTICITY

Authors: DAVID SÁNCHEZ-GÓMEZ and ISMAEL ARANDA

The following Supporting Information is available for this article:

Figure S1. Experimental design and description of the application of water stress. Watering treatment accounted for two levels (well-water level – WW– and water-stress level –WS–). T_0_ denotes the start of the application of water stress to WS-seedlings.

**
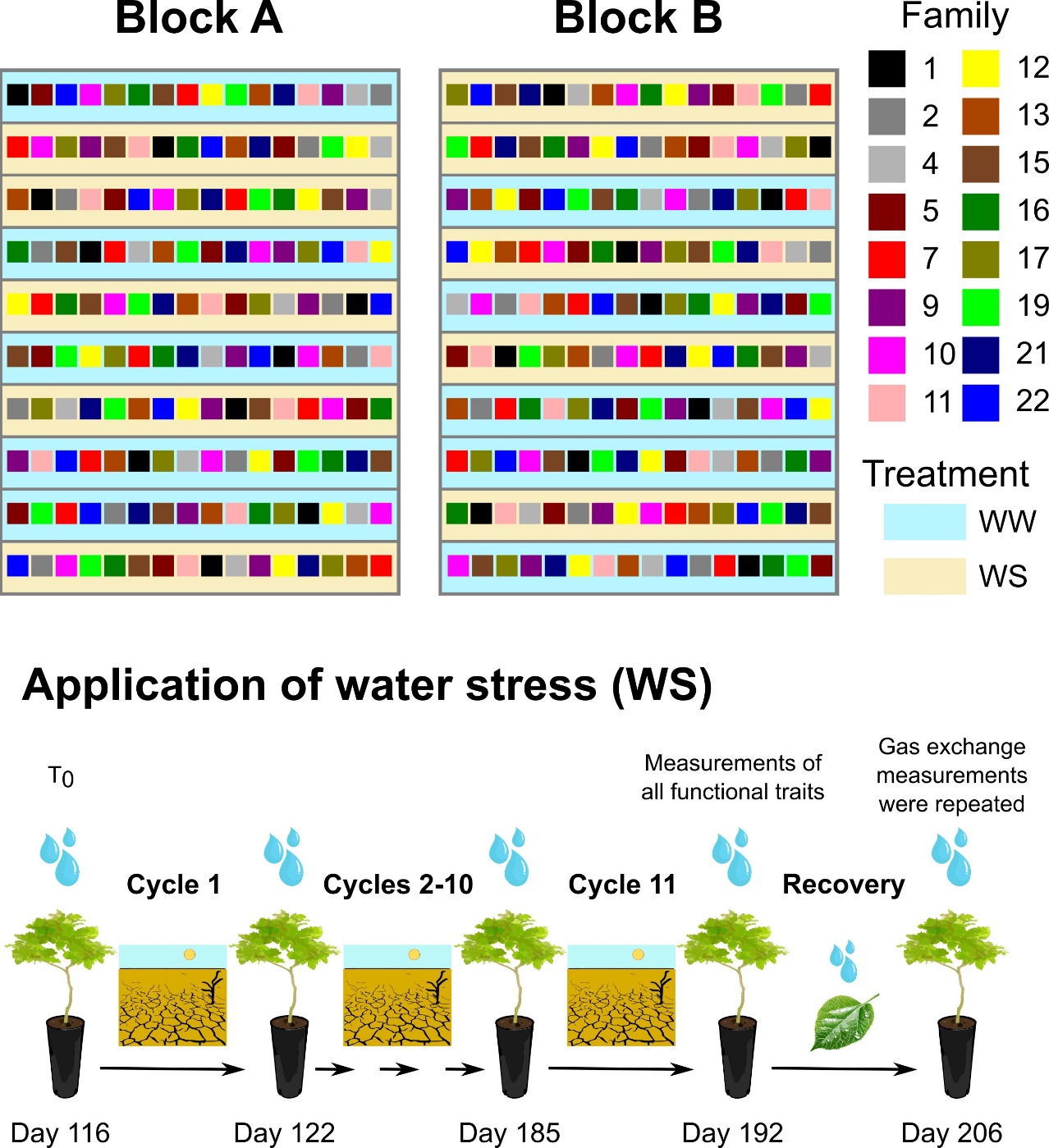
**

Table S1. Indices of phenotypic plasticity to water availability (PP), and post-drought recovery (RE), calculated for each trait and beech family. A value of 0 means complete recovery for RE and lack of plasticity for PP. The letter codes denote homogeneous groups across families

| Family | Index-trait | | | | | | | | |
| --- | --- | --- | --- | --- | --- | --- | --- | --- | --- |
|  | PP-A_area_ | PP-A_mass_ | PP-PNUE | PP-g_wv_ | PP-iWUE | PP-Φ_PSII_ | PP-δ^13^C | PP-δ^15^N | PP-N_m_ |
| 1 | 6.8 x 10^-1^ ab | 6.6 x 10^-1^ ab | 6.8 x 10^-1^ abc | 7.9 x 10^-1^ abcd | 4.3 x 10^-1^ a | 3.4 x 10^-1^ abc | 6.9 x 10^-2^ a | 2.6 x 10^-1^ cde | 1.9 x 10^-2^ ef |
| 2 | 4.8 x 10^-1^ b | 3.7 x 10^-1^ c | 5.2 x 10^-1^ c | 5.9 x 10^-1^ d | 2.6 x 10^-1^ cde | 2.1 x 10^-1^ bc | 4.6 x 10^-2^ abcd | 6.2 x 10^-1^ abcd | 2.5 x 10^-1^ ab |
| 4 | 4.8 x 10^-1^ b | 4.9 x 10^-1^ bc | 6.0 x 101 bc | 6.1 x 10^-1^ cd | 2.9 x 10^-1^ bcde | 2.0 x 10^-1^ c | 4.6 x 10^-2^ abcd | 3.0 x 10^-1^ bcde | 9.2 x 10^-3^ ef |
| 5 | 8.0 x 10^-1^ a | 7.8 x 10^-1^ a | 8.2 x 10^-1^ a | 8.5 x 10^-1^ ab | 3.7 x 10^-1^ abcde | 4.1 x 10^-1^ abc | 6.7 x 10^-2^ ab | 6.4 x 10^-1^ abcd | 8.4 x 10^-2^ cdef |
| 7 | 6.7 x 10^-1^ ab | 6.2 x 10^-1^ abc | 5.8 x 10^-1^ abc | 8.1 x 10^-1^ ab | 4.2 x 10^-1^ ab | 3.1 x 10^-1^ abc | 5.4 x 10^-2^ abcd | 4.9 x 10^-1^ bcd | 3.4 x 10^-2^ def |
| 9 | 7.2 x 10^-1^ ab | 6.7 x 10^-1^ ab | 6.9 x 10^-1^ abc | 8.4 x 10^-1^ ab | 2.2 x 10^-1^ de | 4.4 x 10^-1^ a | 2.8 x 10^-2^ d | 1.1 x 10^-3^ e | 1.2 x 10^-1^ bcd |
| 10 | 6.0 x 10^-1^ ab | 5.0 x 10^-1^ bc | 5.2 x 10^-1^ c | 7.7 x 10^-1^ abcd | 2.8 x 10^-1^ bcde | 3.3 x 10^-1^ abc | 2.6 x 10^-2^ d | 2.1 x 10^-2^ e | 3.6 x 10^-2^ cdef |
| 11 | 6.8 x 10^-1^ ab | 5.8 x 10^-1^ abc | 6.5 x 10^-1^ abc | 8.5 x 10^-1^ ab | 3.4 x 10^-1^ abcde | 3.4 x 10^-1^ abc | 7.0 x 10^-2^ a | 5.3 x 10^-1^ abcd | 6.5 x 10^-2^ cdef |
| 12 | 6.1 x 10^-1^ ab | 5.1 x 10^-1^ abc | 6.2 x 10^-1^ abc | 7.6 x 10^-1^ bcd | 1.9 x 10^-1^ e | 2.8 x 10^-1^ abc | 3.8 x 10^-2^ bcd | 2.4 x 10^-3^ e | 1.1 x 10^-1^ bcdef |
| 13 | 7.5 x 10^-1^ ab | 7.1 x 10^-1^ ab | 7.6 x 10^-1^ ab | 8.6 x 10^-1^ a | 2.0 x 10^-1^ e | 3.8 x 10^-1^ abc | 6.3 x 10^-2^ abc | 5.9 x 10^-1^ abc | 1.1 x 10^-1^ bcdef |
| 15 | 6.8 x 10^-1^ ab | 5.8 x 10^-1^ abc | 6.9 x 10^-1^ abc | 7.9 x 10^-1^ abcd | 3.7 x 10^-1^ abcde | 2.8 x 10^-1^ abc | 5.4 x 10^-2^ abcd | 9.6 x 10^-2^ de | 2.5 x 10^-1^ a |
| 16 | 7.0 x 10^-1^ ab | 6.9 x 10^-1^ ab | 7.2 x 10^-1^ abc | 8.1 x 10^-1^ ab | 3.7 x 10^-1^ abcd | 3.9 x 10^-1^ abc | 6.6 x 10^-2^ ab | 6.8 x 10^-1^ ab | 1.2 x 10^-3^ f |
| 17 | 7.6 x 10^-1^ a | 7.8 x 10^-1^ a | 7.9 x 10^-1^ a | 8.3 x 10^-1^ abc | 4.2 x 10^-1^ abc | 4.1 x 10^-1^ ab | 6.6 x 10^-2^ ab | 1.5 x 10^-1^ bcde | 1.0 x 10^-1^ bcde |
| 19 | 7.6 x 10^-1^ a | 7.4 x 10^-1^ ab | 7.5 x 10^-1^ ab | 8.3 x 10^-1^ abc | 3.0 x 10^-1^ bcde | 3.2 x 10^-1^ abc | 6.0 x 10^-2^ abc | 8.8 x 10^-1^ a | 1.6 x 10^-1^ bc |
| 21 | 5.6 x 10^-1^ ab | 5.7 x 10^-1^ abc | 6.2 x 10^-1^ abc | 7.9 x 10^-1^ abcd | 4.5 x 10^-1^ a | 2.8 x 10^-1^ abc | 6.7 x 10^-2^ a | 7.6 x 10^-1^ ab | 1.1 x 10^-1^ bcd |
| 22 | 6.0 x 10^-1^ ab | 5.9 x 10^-1^ abc | 6.3 x 10^-1^ abc | 7.5 x 10^-1^ bcd | 2.9 x 10^-1^ bcde | 3.0 x 10^-1^ abc | 3.2 x 10^-2^ cd | 7.8 x 10^-1^ a | 1.5 x 10^-1^ bc |

Table S1. Continued

| Family | Index-trait | | | | | | | | |
| --- | --- | --- | --- | --- | --- | --- | --- | --- | --- |
|  | PP-C_m_ | PP-SLA | PP-RGR_T1_ | PP-RGR_T2_ | PP-RGR_Tot_ | RE-A_area_ | RE-g_wv_ | RE-iWUE | RE-Φ_PSII_ |
| 1 | 4.4 x 10^-2^ a | 3.9 x 10^-2^ bcde | 2.8 x 10^-1^ abc | 7.6 x 10^-3^ g | 1.9 x 10^-1^ bcde | 9.2 x 10^-2^ abc | 1.6 x 10^-1^ ab | 7.5 x 10^-2^ cd | 5.0 x 10^-2^ abcdef |
| 2 | 1.6 x 10^-2^ abc | 1.7 x 10^-1^ abc | 1.8 x 10^-1^ bcde | 7.2 x 10^-4^ g | 6.0 x 10^-2^ e | 7.1 x 10^-2^ bcd | 1.6 x 10^-1^ ab | 9.4 x 10^-2^ bcd | 2.2 x 10^-2^ bcdef |
| 4 | 1.2 x 10^-2^ bc | 1.3 x 10^-3^ e | 1.6 x 10^-1^ cde | 8.9 x 10^-2^ cdefg | 1.6 x 10^-1^ bcde | 1.1 x 10^-1^ abc | 2.5 x 10^-1^ a | 1.5 x 10^-1^ a | 1.2 x 10^-2^ cdef |
| 5 | 1.3 x 10^-2^ abc | 2.5 x 10^-4^ e | 1.3 x 10^-1^ cde | 2.8 x 10^-1^ a | 2.1 x 10^-1^ abcd | 0 cd | 1.3 x 10^-1^ ab | 1.5 x 10^-1^ a | 0 f |
| 7 | 1.4 x 10^-2^ abc | 6.3 x 10^-2^ abcde | 2.2 x 10^-1^ abcd | 1.9 x 10^-1^ abcdef | 2.1 x 10^-1^ abcd | 0 d | 1.4 x 10^-1^ ab | 1.5 x 10^-1^ a | 0 f |
| 9 | 2.0 x 10^-3^ bc | 1.9 x 10^-1^ ab | 2.3 x 10^-1^ abcd | 2.2 x 10^-1^ abcd | 2.2 x 10^-1^ ab | 7.1 x 10^-2^ bcd | 1.7 x 10^-1^ ab | 1.0 x 10^-1^ bc | 7.7 x 10^-2^ abcde |
| 10 | 7.3 x 10^-4^ bc | 1.3 x 10^-1^ abcd | 2.1 x 10^-1^ bcd | 4.9 x 10^-4^ g | 9.7 x 10^-2^ cde | 8.2 x 10^-2^ abc | 1.2 x 10^-1^ b | 4.0 x 10^-2^ cd | 8.2 x 10^-2^ abcde |
| 11 | 1.3 x 10^-2^ abc | 6.2 x 10^-2^ bcd | 1.4 x 10^-1^ cde | 1.5 x 10^-1^ abcdefg | 1.6 x 10^-1^ bcde | 0 cd | 1.3 x 10^-1^ ab | 1.4 x 10^-1^ ab | 0 f |
| 12 | 3.1 x 10^-2^ ab | 2.2 x 10^-1^ a | 1.7 x 10^-1^ cde | 1.5 x 10^-2^ defg | 1.7 x 10^-1^ bcde | 1.6 x 10^-1^ ab | 1.5 x 10^-1^ ab | 0 d | 8.4 x 10^-2^ abcde |
| 13 | 9.5 x 10^-3^ bc | 1.3 x 10^-1^ abc | 2.8 x 10^-1^ abcd | 2.3 x 10^-1^ abc | 2.2 x 10^-1^ ab | 1.0 x 10^-1^ abc | 2.1 x 10^-1^ ab | 1.2 x 10^-1^ abc | 1.1 x 10^-1^ abcd |
| 15 | 1.0 x 10^-4^ c | 9.6 x 10^-2^ abcd | 1.3 x 10^-1^ de | 6.0 x 10^-2^ cdefg | 9.1 x 10^-2^ de | 5.7 x 10^-2^ bcd | 1.7 x 10^-1^ ab | 1.2 x 10^-1^ abc | 3.5 x 10^-2^ abcdef |
| 16 | 2.0 x 10^-2^ abc | 1.3 x 10^-1^ abcd | 2.0 x 10^-1^ bcde | 2.9 x 10^-1^ a | 2.1 x 10^-1^ abcd | 1.7 x 10^-1^ ab | 2.4 x 10^-1^ a | 9.0 x 10^-2^ bcd | 1.5 x 10^-1^ abc |
| 17 | 1.5 x 10^-2^ abc | 2.2 x 10^-2^ cde | 3.9 x 10^-1^ a | 7.8 x 10^-3^ cdefg | 2.3 x 10^-1^ ab | 1.8 x 10^-1^ a | 1.9 x 10^-1^ ab | 9.0 x 10^-3^ d | 1.6 x 10^-1^ ab |
| 19 | 1.3 x 10^-2^ abc | 3.3 x 10^-2^ cde | 3.1 x 10^-1^ ab | 1.3 x 10^-1^ abcdefg | 2.8 x 10^-1^ a | 0 cd | 1.1 x 10^-1^ b | 1.2 x10^-1^ abc | 0 f |
| 21 | 1.2 x 10^-2^ abc | 9.2 x 10^-3^ de | 7.3 x 10^-3^ e | 2.7 x 10^-1^ ab | 1.3 x 10^-1^ cde | 1.3 x 10^-1^ abc | 1.8 x 10^-1^ ab | 5.6 x 10^-2^ cd | 1.8 x 10^-1^ a |
| 22 | 2.5 x 10^-2^ ab | 5.1 x 10^-2^ abcde | 2.4 x 10^-1^ abcd | 2.1 x 10^-1^ abcde | 1.7 x 10^-1^ bcde | 2.6 x 10^-2^ bcd | 1.3 x 10^-1^ ab | 9.9 x 10^-2^ bc | 2.1 x 10^-2^ cdef |

Figure S2. Soil volumetric water content throughout the experiment for well-watered seedlings –WW– and water-stressed seedlings –WS–. The points and error bars represent mean ± SE. T_0_ denotes the start of the application of water stress to WS-seedlings.. The timing of several water stress cycles and post-drought recovery are indicated.


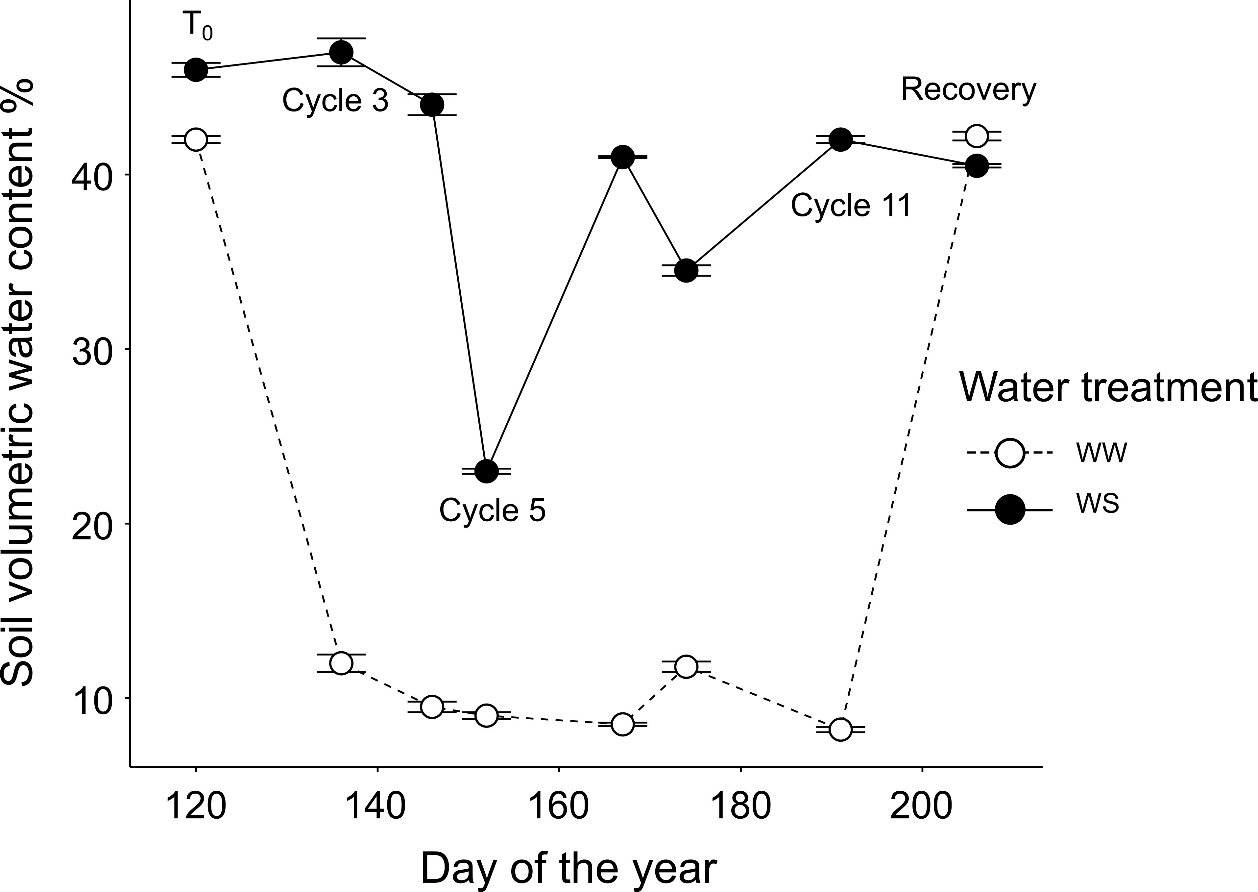


Figure S3. Scatterplot representing the relationship between stomatal conductance to water vapour (g_wv_) and predawn water potential (Ψ_pd_) for all the beech families studied.


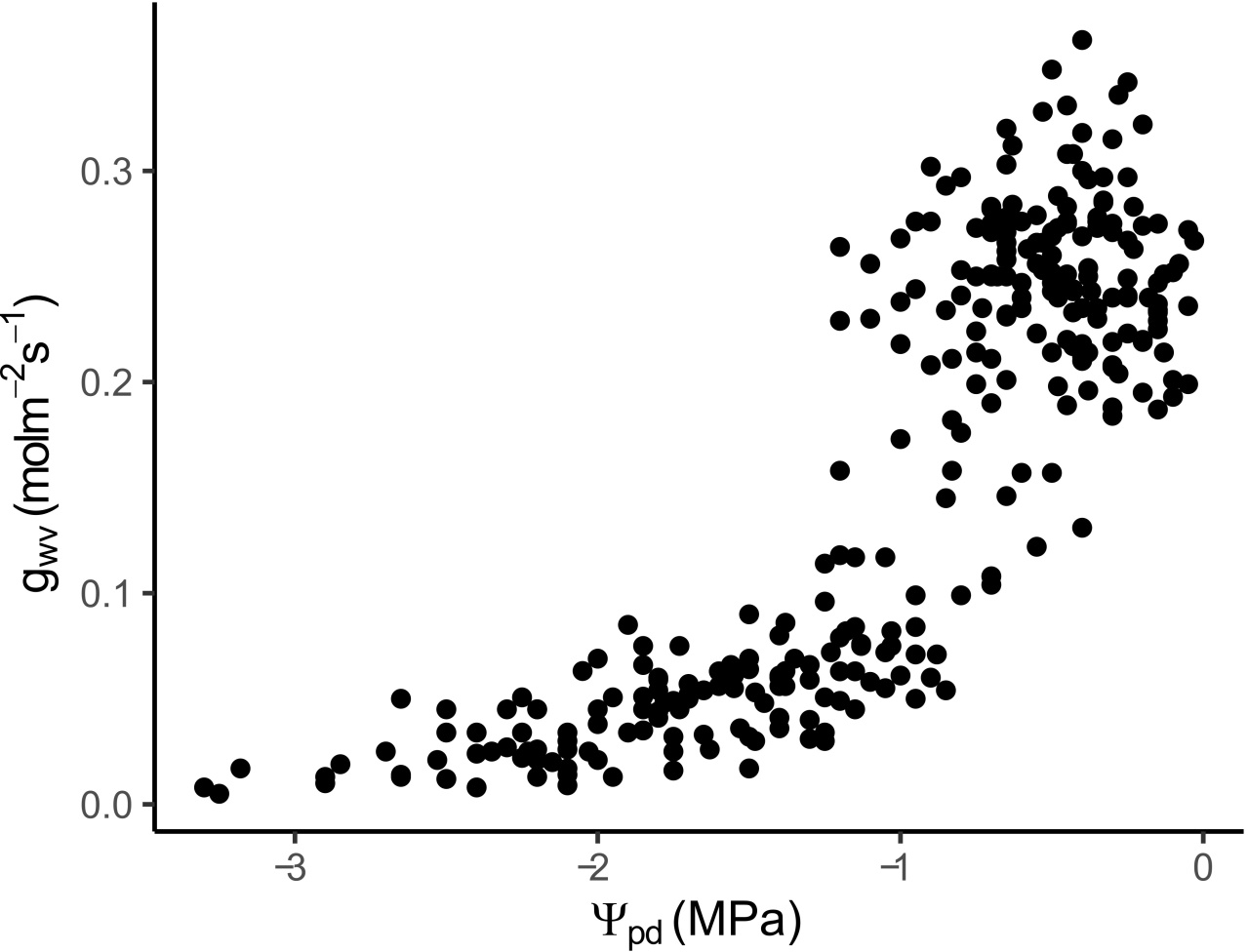


Figure S4. Bivariate relationship between plasticity of A_mass_ (PP-A_mass_) and plasticity of stem basal growth at different time intervals (RGR_T1_, RGR_T2_ and RGR_Tot_).


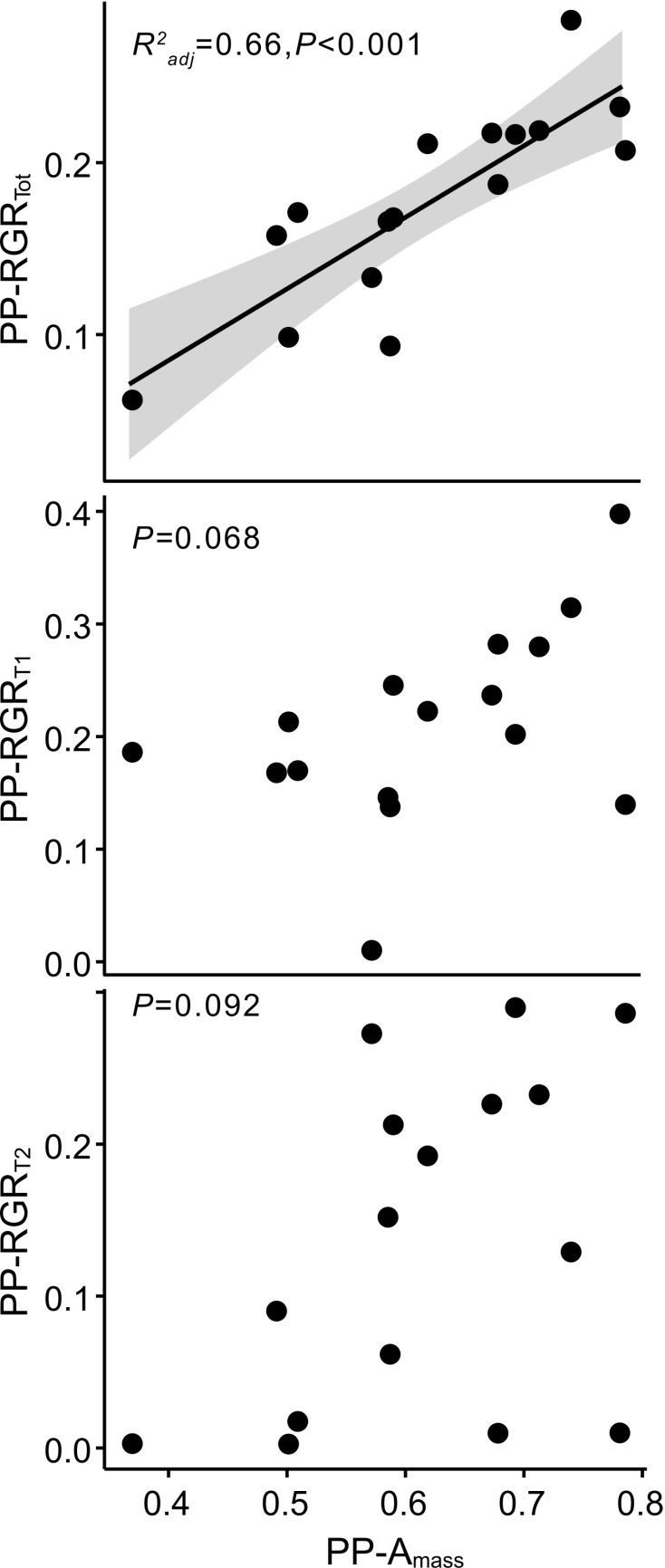


Figure S5. Bivariate relationships of post-drought recovery of gas exchange traits with phenotypic plasticity of the same traits and the values of those traits under water stress.


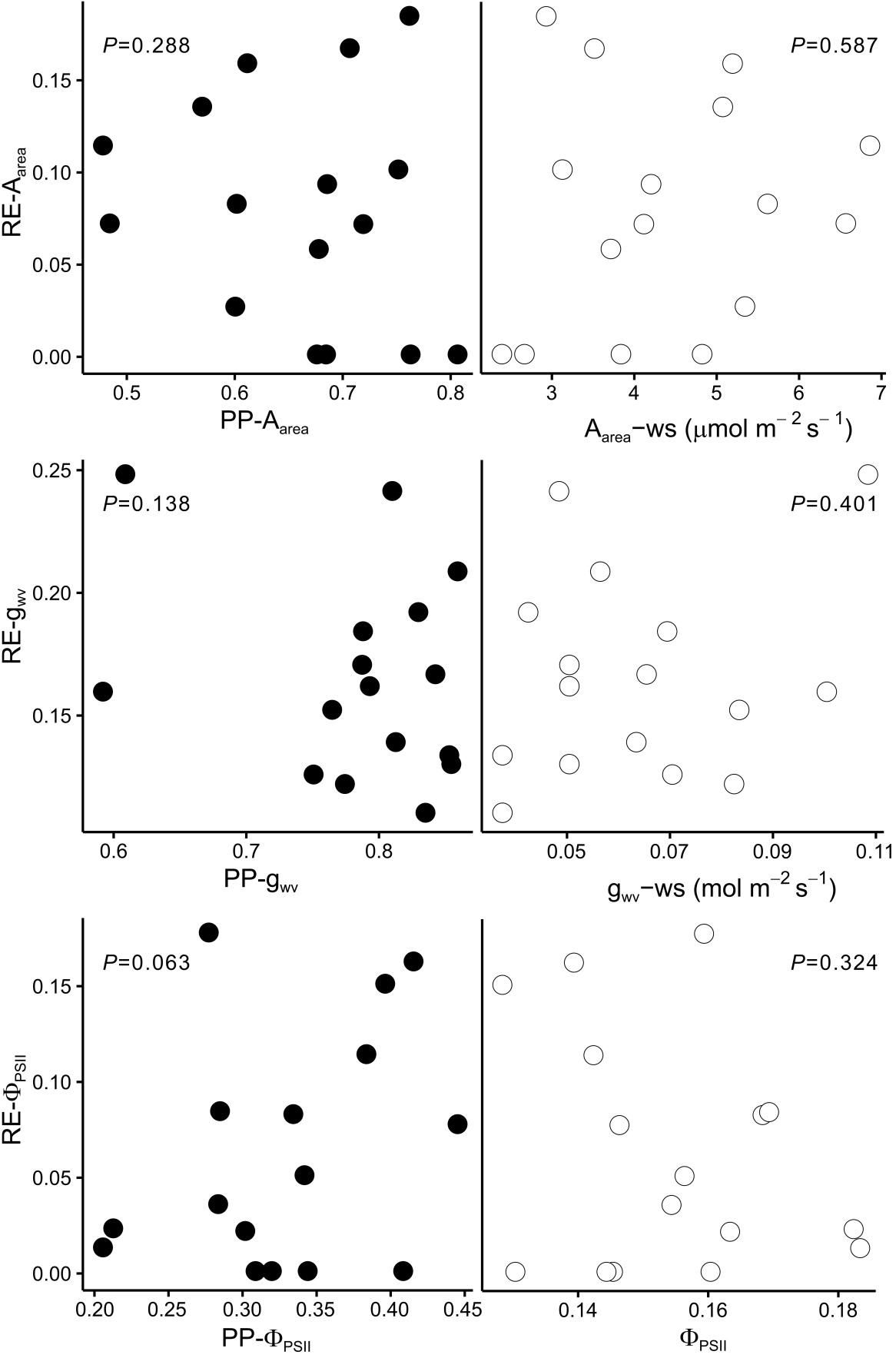

Supplement: supplementary-dsg-ia-revision_tpae107 [file supplementary-dsg-ia-revision_tpae107.docx]
